# Coexistence trend contingent to Mediterranean oaks with different leaf habits

**DOI:** 10.1002/ece3.2840

**Published:** 2017-03-23

**Authors:** Arianna Di Paola, Alain Paquette, Antonio Trabucco, Simone Mereu, Riccardo Valentini, Francesco Paparella

**Affiliations:** ^1^ IAFES DivisionEuro‐Mediterranean Center on Climate Change (CMCC)ViterboItaly; ^2^ Centre for Forest Research (CFR)Université du Québec à MontréalMontréalQCCanada; ^3^ IAFES DivisionEuro‐Mediterranean Center on Climate Change (CMCC)SassariItaly; ^4^ Department of Science for Nature and Environment Resources (DipNET)University of SassariSassariItaly; ^5^ Department for Innovation in BiologicalAgro‐Food and Forest SystemsUniversity of TusciaViterboItaly; ^6^ Strategic Council MemberEuro‐Mediterranean Center for Climate Change (CMMC)ViterboItaly; ^7^ Division of SciencesNew York University Abu DhabiAbu DhabiUnited Arab Emirates; ^8^ Department of Mathematics, University of SalentoLecceItaly

**Keywords:** coexistence model, complementarity, evapotranspiration, Mediterranean diversity, niches partitioning, Quercus spp., species distributions

## Abstract

In a previous work we developed a mathematical model to explain the co‐occurrence of evergreen and deciduous oak groups in the Mediterranean region, regarded as one of the distinctive features of Mediterranean biodiversity. The mathematical analysis showed that a stabilizing mechanism resulting from niche difference (i.e. different water use and water stress tolerance) between groups allows their coexistence at intermediate values of suitable soil water content. A simple formal derivation of the model expresses this hypothesis in a testable form linked uniquely to the actual evapotranspiration of forests community. In the present work we ascertain whether this simplified conclusion possesses some degree of explanatory power by comparing available data on oaks distributions and remotely sensed evapotranspiration (MODIS product) in a large‐scale survey embracing the western Mediterranean area. Our findings confirmed the basic assumptions of model addressed on large scale, but also revealed asymmetric responses to water use and water stress tolerance between evergreen and deciduous oaks that should be taken into account to increase the understating of species interactions and, ultimately, improve the modeling capacity to explain co‐occurrence.

## 1 Introduction

The Mediterranean region holds an extraordinary diversity of species because of the multitude of different habitats it contains (Cody, 1986). These habitats, commonly established as forests, woodlands, savannas, grasslands and wetlands (Friedl et al., 2002), are highly distinctive, collectively harboring 10% of the Earth's plant species, including many rare and endemic plants (Cody, 1986; Cowling, Rundel, Lamont, Kalin Arroyo, & Arianoutsou, 1996). The native arboreal vegetation of the warmest zones of the Mediterranean climate is typically evergreen sclerophyllous, adapted to cope with summer water stress and infertile soils (Gasith & Resh, 1999). Where the climate is characterized by relatively higher precipitation and lower winter temperature, the characteristic vegetation is then dominated by deciduous trees (Chabot & Hicks, 1982).

Scattered in the Mediterranean region are places where sclerophyllous evergreen and deciduous species co‐occur (Bonfil, Cortés, Espelta, & Retana, 2004; Damesin, Rambal, & Joffre, 1998; Mediavilla & Escudero, 2003), which is regarded as one of the distinctive features of the Mediterranean biome (Baldocchi et al., 2010; Givnish, 2002; Noce, Collalti, Valentini, & Santini, 2016).

Literature supports a correspondence between evergreen and deciduous leaf traits and both water use and water stress tolerance among oaks: evergreen oaks are less vulnerable to drought‐induced embolism than deciduous ones (Choat et al., 2012; Niinemets, 2001); For instance, Choat et al. (2012) have drawn together data on the vulnerability of the transport system to drought‐induced embolism for a large number of woody species, showing a distinctive global convergence in the vulnerability of deciduous oaks with respect to the evergreen.

By contrast, Niinemets and Valladares (2006) showed a negative correlation between drought and waterlogging tolerance among oaks in the Northern Hemisphere (Niinemets & Valladares, 2006) and it is possible that waterlogging limits the occurrence of evergreens in the more humid areas (Glenz, Schlaepfer, Iorgulescu, & Kienast, 2006; Niinemets & Valladares, 2006; Rodríguez‐González, Stella, Campelo, Ferreira, & Albuquerque, 2010), albeit a comprehensive knowledge about waterlogging effects on deciduous and evergreen oaks is still lacking.

Deciduous and evergreen oaks also differ in leaf mass per area (LMA)(Cornelissen, Diez, & Hunt, 1996), which represents the investment in carbon made by the species for a unit leaf area. Generally, deciduous trees compensate for a shorter growing season by producing leaves that have a low LMA, high photosynthetic/transpiration rates (Baldocchi et al., 2010; Chabot & Hicks, 1982; Schulze, 2002) and high nitrogen content. Conversely, Mediterranean evergreen species have a longer growing season, produce leaves characterized by lower nutrient availability and higher LMA. This confers them a greater resistance to drought at the expense of lower photosynthetic and transpiration rates (Costa‐Saura, Martínez‐Vilalta, Trabucco, Spano, & Mereu, 2016; Poorter, Niinemets, Poorter, Wright, & Villar, 2009; Tyree & Cochard, 1996), albeit on yearly average their primary gross production is similar to that of deciduous oaks (Baldocchi et al., 2010).

In a previous work (Di Paola, Valentini, & Paparella, 2012) we developed a mathematical model to represent the co‐occurrence of sclerophyllous evergreen oaks (*Q. ilex* and *Q. suber*) and deciduous oaks (dominated by a typical endemism of *Q. cerris* and *Q. frainetto*, as well as *Q. robur*) in two sites of Tyrrhenian plain forests (western coast of Italy), where the abundance of edaphic water, despite the warm Mediterranean climate, allows the co‐occurrence of these species.

The model describes the competition for water between two species, in our case evergreen and deciduous oak groups, that have different niches, that is, different water use (transpiration rate) and water stress tolerance (drought and waterlogging) but similar growth rate, on yearly averages.

According to the model, in the lower end of suitable soil water content range evergreen oaks are able to competitively exclude deciduous oaks, while in the upper end the opposite happens. When the environmental conditions allow an intermediate range of soil water content, the different groups attain a stable coexistence equilibrium. The mathematical analysis shows that the equilibrium of coexistence results from the long‐term interplay of different transpiration rates and water stress tolerances between evergreen and deciduous oaks: if, in the coexistence state, some deciduous oaks were replaced with evergreen oaks, the whole community would have a slightly smaller overall evapotranspiration, leading to a slightly larger soil water content, which, in turn, would let the deciduous be slightly more competitive than the other group, up until the equilibrium is re‐established. If, instead, some of the evergreen oaks were replaced by deciduous oaks, a slightly larger evapotranspiration would lead to slightly lower soil water content, and thus a competitive advantage for the evergreens group, until the equilibrium is attained again.

Thus, at intermediate interval of suitable soil water content, the model admits a stable coexistence between evergreen and deciduous oak groups thanks to a stabilizing mechanism resulting from niche differences whereby the intra‐specific effects exceed inter‐specific effects (i.e. each species favor the competitor more than themselves), consistently to the classic coexistence theory (Chesson, 2000; Wilson, 2011).

Here, we show how a simple formal derivation of the model allows to express the criteria for a stable coexistence in a testable form solely linked to forest actual evapotranspiration (ET): the actual evapotranspiration of a forest composed only by evergreen oaks (ET_*E*_) should be lower than that of co‐occurrence of both the groups (ET_CO_), which, in turn, should be lower than that of deciduous oaks community (ET_*D*_). To ascertain whether such a simplified description posses some degree of explanatory power, we compared the model derivation with existing data on oaks distributions and remote sensing derived ET (MODIS product) in a large‐scale survey embracing the western Mediterranean area. To our knowledge this is the first large‐scale assessment (*i*) of co‐occurrence of Mediterranean oaks, while (*ii*) verifying the general conditions for a large‐scale coexistence model.

Despite the potential for the coexistence theory to be simple yet complex enough to resemble reality, coexistence models are generally approached at some defined local area (Cavieres et al., 2014; Chesson, 2000), without further application on regional scales. Conversely, large‐scale data dealing with species interactions and that could be of support to coexistence models are still largely missing (Cavieres et al., 2014). The present study also tried to shorten this gap.

Our findings confirmed the basic assumptions of the model at regional scale, but also revealed asymmetric responses to water use and water stress tolerance between evergreen and deciduous oak groups that should be taken into account to increase the understating of species interactions and, ultimately, improve the modeling capacity to explain oaks co‐occurrence.

## 2 Methods

### 2.1 Model overview

Here we give a brief overview of the model, which background is useful to understand the mechanism allowing for a stable coexistence. It was presented in detail in (Di Paola et al., 2012), and we refer the interested reader to our previous paper for the stability analysis and any further details.

The model represents the competition for water between two species, in our case evergreen and deciduous oak groups, who assume similar growth rate but different water use and water stress tolerance on yearly averages, tracing conditions for a stable coexistence (see Chesson (2000) and Wilson (2011) for an overview of the coexistence theory). Hereinafter, the word “co‐occurrence” will be used when referring to the evidences from spatial data of the presence of both groups in the same location, while the term “coexistence” refers to the homonymous stable equilibrium of the model.

The model's equations are:

(1) $$\dot{E}\;=\;H\left(W\right)E\;-\;k_{E}\left(D\;+\;E\right)E$$

(2) $$\dot{D}\;=\;F\left(W\right)D\;-\;k_{D}\left(D\;+\;E\right)D$$

(3) $$\dot{W}\;=\;S\left(W\right)\;-\;T_{E}\left(W\right)E\;-\;T_{D}\left(W\right)D$$

where the *E* and *D* within the equations represent the biomass density (mass per unit area) of evergreen and deciduous oak groups, respectively, and *W* is the soil water content. The dot above the variables denotes differentiation with respect to time. The functions *F* and *H* are the net growth rates of deciduous and evergreen, respectively, and, over the realistic range of soil water content of Mediterranean forests, they are assumed to be a growing function of *W*. *F* and *H* differ because scarcity of water is more stressful for deciduous oaks than for evergreen ones, while the opposite happen in the case of water overabundance (Choat et al., 2012; Gellini & Grossoni, 1997; Niinemets & Valladares, 2006; Tyree & Cochard, 1996). Hence, *F* is smaller than *H* at the lower end of the suitable soil water content range, but greater at the upper end. The second term in the right‐hand side of (1) and (2) models the competition for space, and describes a mortality rate proportional to the stand total biomass (*D + E*); *k*_*D*_ and *k*_*E*_ are proportionality constants whose numerical values are not very different for the two groups of species *T*_*D*_ and *T*_*E*_ are also growing functions of *W* and represent the water loss by transpiration per unit of biomass. The model assumes *T*_*D*_ smaller than *T*_*E*_ because evergreen oaks are supposed to have higher water use efficiency, while deciduous oaks may reach larger metabolic growth rates at the upper end of the soil water content range but at the expense of higher transpiration (Rodríguez‐González et al., 2010; Schulze, 2002; Chabot & Hicks, 1982).

The function *S* quantifies the algebraic sum of all biomass‐independent sources and sinks of water:

(4) $$S\left(W\right)\;=\;p\;-\;e\;-\;q\left(W\right)$$

where *p* is the precipitation, *e* is the evaporation, and the function *q* models the rate of water losses through run‐off, subsurface lateral flows and percolation.

As shown in (Di Paola et al., 2012) the stability of the model's equilibria does not depend on the numerical details of the functions that appear in it, but only on their broad features as summarized above (e.g. the request that *F* be smaller than *H* at lower values of *W*, but overtake it at higher values of *W*) and their monotonicity properties.

The model admits three meaningful stable equilibria: an equilibrium where the forest is composed only by evergreen oaks, which is stable when the soil water content is low; a coexistence equilibrium, which is stable over an intermediate range of soil water content, and an equilibrium with only deciduous oaks, which is stable at higher water availability. The switch between these equilibria (i.e. only evergreen oak group, coexistence, only deciduous oak group) are due to climate‐induced trade‐offs between the inter‐ and intra‐specific competition that, respectively, limits or enhances a stable coexistence (intra‐specific competition means that a species favors the competitor more than oneself). Thus, when the climate regime leads to the lower end of the soil water content range the evergreen oaks are favored thank to their ability to tolerate drought, grow with higher water use efficiency, and, thus, are able to competitively exclude the deciduous. At the upper end the opposite happens. Over intermediate interval of suitable soil water content range, the intra‐specific competition effects exceed the inter‐specific ones, yielding a stable coexistence around an homeostatic equilibrium of *W* (see Appendix S1 for a proof). The dynamic allowing a stable coexistence depends on the long‐term interplay of transpiration rates and water stress tolerances of the two groups: if, in the coexistence state, some deciduous oaks were replaced with evergreen ones, the whole community would have a slightly smaller overall evapotranspiration, leading to a slightly larger soil water content, which, in turn, would let the deciduous be slightly more competitive than evergreen oaks, up until the equilibrium is re‐established. If, instead, some of the evergreen oaks were replaced by deciduous ones, a slightly larger evapotranspiration would lead to slightly lower soil water content, and thus a competitive advantage for the evergreen group, until the equilibrium is attained again.

An interesting and testable prediction of the model is shown in Appendix S1 and leads to the following chain of inequalities:

(5) $${\text{ET}}_{E}<{\text{ET}}_{\mathrm{CO}}<{\text{ET}}_{D}$$

where ET is the evapotranspiration rate of a state of stable equilibrium, and the subscripts *E*,* D* and CO refer, respectively, to the equilibrium with only evergreen oaks, deciduous oaks, and the coexistence of both the groups. Thus the model states that the evapotranspiration of a forest in a state of stable coexistence should be sandwiched between the evapotranspiration of a forest composed only by evergreen oaks and that of a forest composed only by deciduous oaks. This is an unambiguous statement of the model that has the strong advantage of be linked uniquely to the evapotranspiration variables, and thus suitable for a direct comparison with data.

To understand whether the inequalities (5) are reflected into the actual distribution of Mediterranean oaks, we examined existing spatial data on oak distributions and forest evapotranspiration, at ca. 1 km spatial scale. This is the highest resolution at which large‐scale requested data were available, and reflects the common level of spatial detail achievable for most regional studies on species distribution. Because the model was originally approached at local scale, as preliminary step we had to verify that the basic assumption on niche differences (different water use and water stress tolerance) at the spatial scale here addressed still made sense. This was accomplished by looking at evergreen and deciduous oaks niche segregations with respect to both actual and potential evapotranspiration.

### 2.2 Data source

Oak species distributions are taken from the European Commission's Joint Research Centre (JRC), which provides a geodatabase at 1 km resolution of tree distribution for 115 different tree species, expressed as percentage of land cover (Köble & Seufert, 2001). The sum of the percentages of the individual tree species returns the percentage of land covered by forest. The database includes 30 European countries and currently represents the most accurate and up‐to‐date large‐scale dataset of tree species distributions in Europe, although some species and countries are still not included.

The MODIS MOD‐16 Global Terrestrial Evapotranspiration dataset (Mu, Zhao, & Running, 2011) provides the mean annual water flux lost through evapotranspiration (mm/year) at 30 arc sec resolution (~1 km), covering the time period 2000–2011 (i.e. 12 geotiff maps). From the MODIS dataset, we computed the time‐averaged actual evapotranspiration (ET) shown in Fig. S1. The evapotranspiration algorithm is based on the Penman‐Monteith equation whose formulation includes parameters of existing land surface conditions and plant canopy development, available from other MODIS products. Mu et al. (2011) reported that magnitudes and spatial patterns of global ET generally agree with the available in‐situ measurements, reaching a Mean Absolute Bias of 0.31–0.39 mm/day (or 113–140 mm/year).

These datasets (JRC species distribution and MODIS‐ET) both have a spatial resolution of ~1 km^2^, hence our analysis was based on the same spatial resolution.

The mean annual Global Potential Evapotranspiration (PET) dataset, estimated according to the Hargreaves equation (Hargreaves, 1994) for the time period 1960–2000 was provided by the CGIAR‐CSI consortium (Zomer, Trabucco, Bossio, van Straaten, & Verchot, 2008). PET measures the ability of the atmosphere to remove water through evapotranspiration processes and strongly depends on climatic drivers. The PET dataset has the same spatial resolution as the MODIS evapotranspiration dataset.

Other ancillary datasets were: mean annual precipitation (P), covering the time period 1960–2000, provided by WorldClim (Hijmans, Cameron, Parra, Jones, & Jarvis, 2005) at a resolution also of 30 arcsec (~1 km); MODIS global land cover map (17 land cover classes) at 1‐km spatial resolution (Friedl et al., 2002).

### 2.3 Data analysis

From the JRC dataset we selected the distributions of the major evergreen oaks (*Q. ilex*,* Q. coccifera*,* Q. suber*, and *Q. rotundifolia)* and deciduous oaks (*Q. cerris*,* Q. frainetto*,* Q. pubescens* and *Q. robur)*. We also looked at the species *Q. trojana*,* Q. pyrenaica* and *Q. faginea* but regarded and discussed them separately as *semi‐deciduous*, due to their mean leaf life‐span of 5–12 months (Gellini & Grossoni, 1997; Villar & Merino, 2001) which is intermediate between those of evergreen and deciduous.

Data on the distribution of species were converted to the same projection as the ET data, that is to geographical coordinates (WGS84 geoid) with a resolution of 30 arcsec. The boundary of the study includes Mediterranean forests, mostly in the Western Euro‐Mediterranean area, characterized by hot‐dry summers and rainy winters (see Köppen‐Geiger climate classification *Csa* and *Csb* (Kottek, Grieser, Beck, Rudolf, & Rubel, 2006; Santini & di Paola, 2015) for further details).

The aggregation of the species according to their leaf habit into the broad categories of deciduous and evergreen groups allowed us to identify the forested areas covered by a pure group (see Figs S2 and S3) as well as areas of co‐occurrence (see Fig. S4), quantified by the following index:

$$\mathrm{CO}=\frac{\left|O_{D}+O_{E}\right|-\left|O_{D}-O_{E}\right|}{O_{D}+O_{E}}$$

where *O*_*E*_ and *O*_*D*_ stand for the occurrence of evergreen and deciduous oak group, respectively, and CO for their co‐occurrence.

Note that the index CO ranges between 0 and 1. If CO = 1, then there is equal occurrence (50%–50%) of the two groups, while CO = 0 expresses total dominance of one of the two groups.

To minimize the probability of bias we selected only grid cells with oaks presence exceed 90%.

As we were interested to look at the group distributions with respect to the climatic variables (PET, ET and P), we singled out three subsets from each climatic dataset: areas where are found 1) only the deciduous group 2) only the evergreen group, and 3) the co‐occurrence. For each subset we used the 10th, 25th, 50th, 75th and 90th percentiles to draw box plots and evaluate the interquartile range (IQR). Moreover, the numerical abundance of data points (always more than 10^3^) allowed us to also do a kernel density estimation (Appendix S2) and draw probability density functions (PDF).

We also estimated the PDF of individual oak species with respect to ET, considering also data points with oaks presence lower than 90% due to a data shortage for single oak species.

Lastly, to assess whether the distributions of ET for each group (*D*,* E* and CO) were drawn from the same distribution we used two nonparametric test: the one‐tailed Mann–Whitney *U* test and the two‐sample Kolmogorov‐Smirnov (KS). The first indicates whether the central tendency of one group was significantly different from the others, while the second indicates these differences between the distribution shapes.

We opted for nonparametric statistics to account for nonnormal distributions. The null hypothesis was that the distribution and central tendency show no difference between functional groups. For each test we report the *p*‐value of the null hypothesis, which may be extremely low, due to the large sample size.

## 3 Results

**Figure 1:**
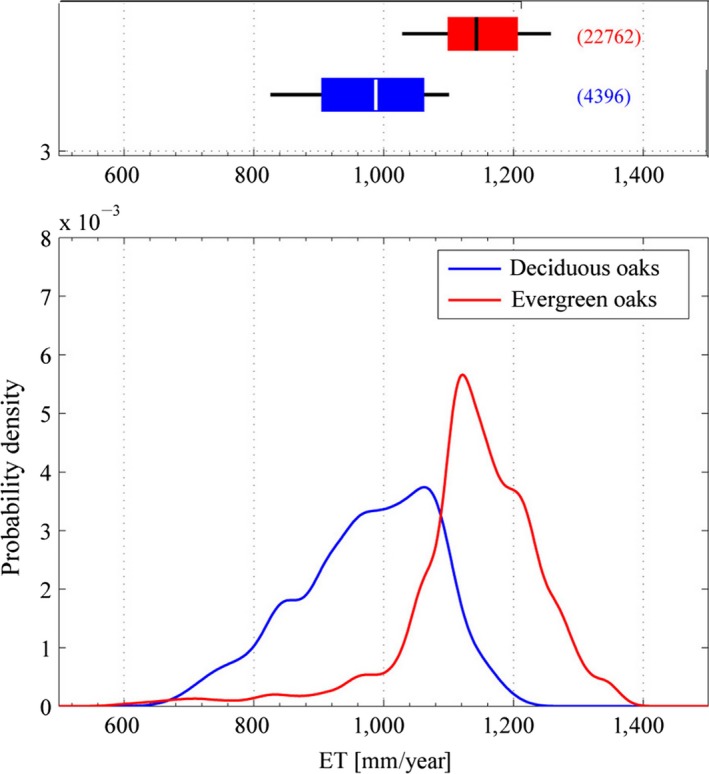
Distributions of deciduous and evergreen oak groups with respect to PET. *Top:* Boxplot. Central lines are the medians, the edges of the boxes are the 25th and 75th percentiles, while whiskers extend to the 10th and 90th percentiles. The brackets show the number of data points; *Bottom:* Probability density functions (PDFs) estimated from data through Eq. (S6)

Differences in water use and water stress tolerance between evergreen and deciduous oaks at the spatial scale here addressed was well supported by large‐scale distributions with respect to both ET and PET. Figure 1 shows that deciduous and evergreen oaks, when they do not co‐occur, tend to fill distinct PET ranges (the IQR of the distributions do not overlap), with evergreen oaks mostly occurring in relatively warmer and dryer regions, reflecting a different water stress tolerance. Nonparametric tests showed a significant difference between PET distributions of the two groups (*U = *3.4 × 10^10^ *;* KS* *= 0.55; both with *p *< 1 *× *10^−15^, see Table S2 for pairwise comparisons).

**Figure 2:**
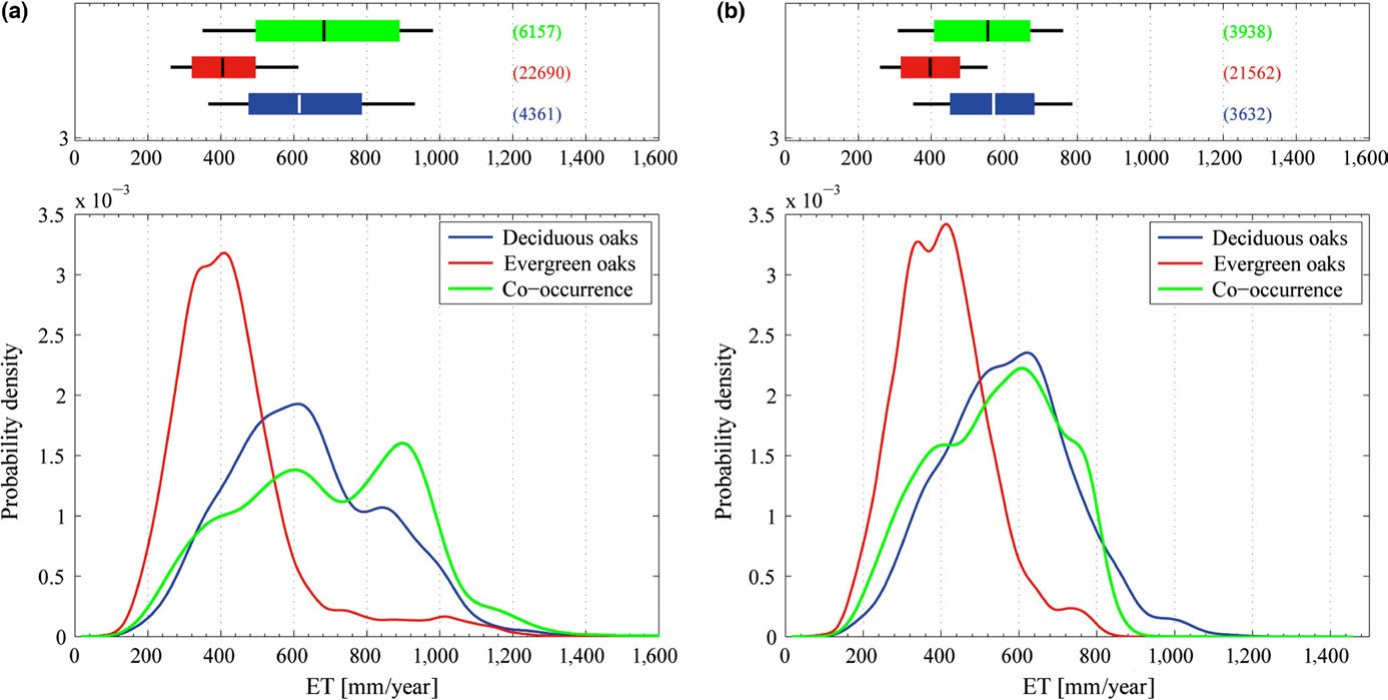
Distributions of evergreen and deciduous oak groups and their co‐occurrence (a) with respect to the actual evapotranspiration (ET); (b) with respect to ET but excluding data where ET > P. *Top*: Boxplot (same as Figure 1); *Bottom*: Probability density functions estimated from data through Eq. (S6)

Probability density curves of evergreen and deciduous oak groups with respect to ET are shown in Figure 2. Most of the sites having only evergreen oaks (cover > 90%) occurred in a narrower range of ET (IQR = 166 mm/year) compared to sites hosting only deciduous (IQR = 302 mm/year), reflecting a different water use between the two groups. The medians (404 and 614 mm/year, for evergreens and deciduous, respectively) were separated by over 200 mm/year, and there was a slight overlap of the IQRs (see Table S1 for further details). The distributions differed markedly at ET larger than 600 mm/year, where evergreen oaks were unlikely to be found. Nonparametric tests always rejected the null hypothesis of no difference between the distributions (*U* = 2.7 × 10^10^ and KS = 0.49, both with *p *< 10^−15^).

Co‐occurrence had the broadest PDF (Figure 2a), overlapping mostly that of the deciduous, and, partially, also that of evergreen oaks. The KS test showed a significant difference between the distribution of co‐occurrence and those of evergreen and deciduous oaks (*p *< 10^−15^, see Table S2 for all pairwise comparison). The median and the IQR of co‐occurrence (684 and 385 mm/year, respectively) were slightly but significantly higher than those of deciduous oaks (614 and 302 mm/year respectively). In Figure 2a the distribution of co‐occurrence is distinctly bi‐modal with a sharp peak at ca. 900 mm/year emerging from a broader distribution peaking at ca. 600 mm/year, suggesting the concomitance of two distinct processes. The peak at ca. 900 mm/year was given by the typical co‐occurrence of *Q. Ilex* and *Q. cerris* and *Q. ilex* and *Q. pubescens* in Italy and France along the Tyrrhenian coast.

Overall, for ET values higher than ca. 870 mm/year, the probability of co‐occurrence (27.7%) exceeded that of finding only deciduous species (15.2%) or only evergreen species (4.1%, see Table S1).

**Figure 3:**
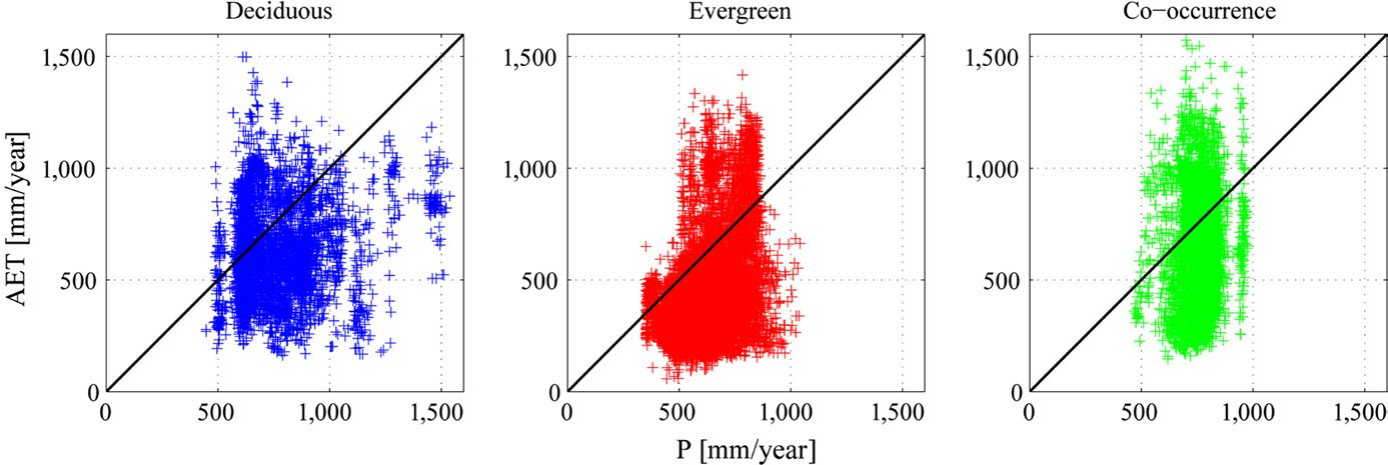
Scatter plot between P (from WorldClim database) and ET (MODIS), considering only data points with percentage of forested land cover ≥90%. The black line is the bisector

Further analyses involving P and ET revealed that about 28%, 9% and 40% of the deciduous, evergreen and co‐occurrence groups, respectively, were characterized by an estimated ratio ET/P greater than 1 (Figure 3, see also Fig. S5 for the location of data having ratio ET/P > 1).

These data points could represent particular topographical conditions (e.g. presence of lateral inflows), or systematic underestimations of rain‐gauge measurements and data mismatches. When these pixels were removed from the analysis (Figure 2b), the results changed remarkably: the unexpected bump in the PDF of co‐occurrence, located between 800 and 1,000 mm/year, and the long tail in the PDF of the evergreen species, both disappeared. For ET values higher than ca. 870 mm/year, the probability of finding only evergreen species or co‐occurrence became <0.01% (see Table S1). The median of co‐occurrence (536 mm/year, Figure 2) sits within the medians of the evergreen and deciduous groups (391 and 540 mm/year, respectively, See Table S1), with the PDF still overlapping mostly that of the deciduous, and only partially that of the evergreen.

Nonparametric tests between the groups always rejected the null hypothesis with high levels of significance (*p *< 10^−7^). In particular, the Mann–Whitney test showed that the central tendency of the evergreen was significantly smaller than that of co‐occurrence, which in turn was significantly smaller than that of the deciduous (see Table S2 for all pairwise comparisons), as suggested by inequalities (5).

**Figure 4:**
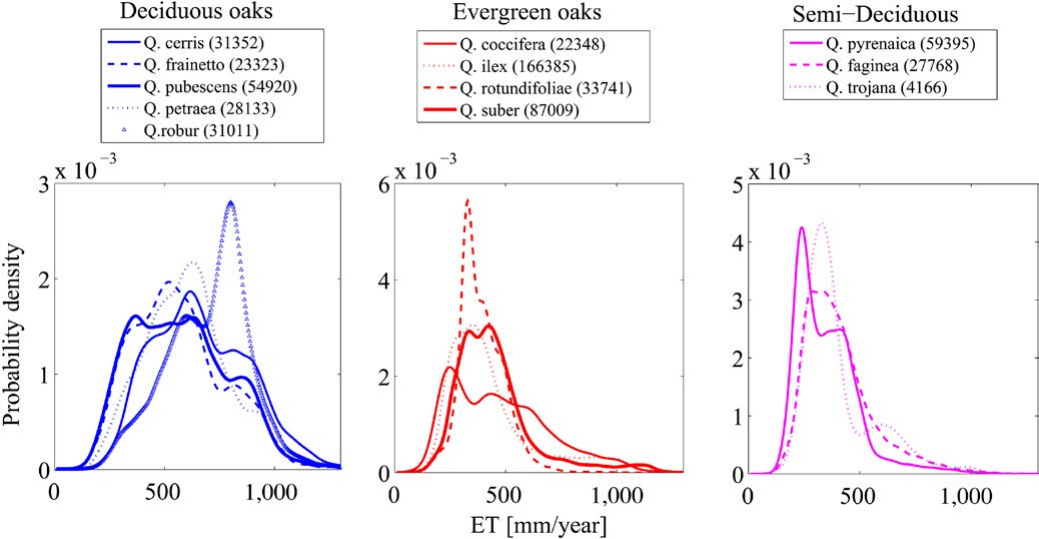
Probability density functions of deciduous (a), evergreen (b) and semi‐deciduous oaks (c) with respect to the ET. PDFs estimated according to Eq. (S6). The brackets show the number of data points

Lastly, Figure 4 shows the probability density of single oak species as a function of ET (cover 0–100% due to shortage of data points), regardless of whether there is co‐occurrence or not.

Semi‐deciduous oaks (Figure 4) revealed quite similar ET distributions with evergreen oaks (medians: 329, 367, 415 mm/year for *Q. faginea*,* Q. pyrenaica* and *Q. trojana*, respectively); Similarities between semi‐deciduous and evergreen oaks were further supported by computing the PDFs resulting from (*i*) including the semi‐deciduous into the group of deciduous oaks (Fig. S6*a*) or (*ii*) into the group of evergreen oaks (Fig. S6*b*). In the first case (Fig. S6*a*), semi‐deciduous oaks mostly affected the distributions of the deciduous group, and that of co‐occurrence at low ET. Instead, the second case (Fig. S6*b*) showed no appreciable difference in comparison with the results shown in Figure 2a, suggesting that at the spatial scale of ca. 1 km semi‐deciduous may be assimilated to the evergreen.

## 4 Discussion

The underlying principle of the model is that differences in water use and water stress tolerance between evergreen and deciduous oak groups allow their coexistence at intermediate interval of soil water content, overcoming the principle of competitive exclusion that, by contrast, prevails at the lower and upper end of suitable soil water content range. The assumption of different water use and water stress tolerance between the two groups was originally supported by literature (Chabot & Hicks, 1982; Baldocchi et al., 2010; Choat et al., 2012; Tyree & Cochard, 1996; Niinemets, 2001; Niinemets & Valladares, 2006) and was also confirmed by analyzing JRC species distribution data in relation to water fluxes defined by MODIS‐ET and CGIAR‐PET bioclimate data: at the scale of ~1 km evergreen and deciduous oak groups had segregated distribution over both the gradient of PET and ET (Figures 1 and 2), reflecting a different water stress tolerance and water use, respectively. We considered this check as a preliminary step to evaluate in order to approach regional scale assessment, because results obtained at different spatial scale might show large variability.

The distribution of co‐occurrence, when odd data (ET/P > 1) were removed, was located in between the single groups (Figure 2b). The central tendency of the evergreens was significantly lower than that of co‐occurrence, which in turn was significantly lower than that of the deciduous, in agreement with inequalities (5). Thus, the interlinkage between bioclimate conditions depicted by ET and the distribution of oaks functional groups shows relevant hints to understand the complex dynamics of co‐occurrence, as indicated by the model. However, the distribution of the co‐occurrence had the broadest PDF that visibly overlaps that of the deciduous group, albeit nonparametric test rejected the null hypothesis of no difference between them.

The CO distribution may reflect asymmetries in the adaptation to water stress and water exploitation strategies of the two groups (Tognetti et al., 1998; Acherar & Rambal, 1992). For example, deciduous oaks may not be able to increase resistance to drought to the same extent to which evergreens may increase their tolerance to waterlogging. Increased resistance to drought implies numerous adaptations which come at a high carbon cost such as low diameter vessels, higher wood density, and high LMA. Increased tolerance to waterlogging on the other hand has a lower cost in terms of carbon investment, such as suberization of the fine roots, more superficial roots and lower max root depth, which would allow evergreens to tolerate humid sites, at least for short periods (Niinemets & Valladares, 2006). Another possible asymmetry could be in the operational range of the water use efficiency, that is, unit of biomass produced per unit water transpired. The occurrence of such asymmetrical responses to abiotic factors is supported by the PDFs of the two groups as a function of PET where the deciduous do not extend to the drier sites as much as evergreens, whereas the evergreens may extend down to the most humid sites.

In disagreement with the prediction of the model, the probability of finding co‐occurrence was larger than that of deciduous at high rates of ET (Figure 2a) when considering also the data points with ET/P > 1.

We cannot exclude that the PDFs of both co‐occurrence and evergreens at higher rates of ET may be due to some inaccuracies in the involved datasets, as suggested by a ratio ET/P > 1. Overall, there are about 5% of data points (more than 600 thousand hectares), having a mean annual ET higher than the mean annual precipitation. We also found occasional mismatches between the land cover classes defined by MODIS (Friedl et al., 2002) and the species distributions provided by the JRC. Most of deciduous and evergreen oak species (ca. 27% and 52%, respectively) fell within the Woody Savannas of MODIS land cover class as expected (see Fig. S7). However, there were also many pixels of deciduous and evergreen groups (ca. 19% and 20%, respectively) falling into odd land cover classes such as Cropland and Grassland, suggesting that the MODIS Land Cover (used in the algorithm for the estimation of ET) and the Tree Distribution Maps do not overlap perfectly, leaving the issue unresolved.

However, a ratio ET/P > 1 is not necessarily an artifact and it has been reported often in areas with other sources of water in the hydrological balance (e.g. lateral flow and water accumulation). In these cases, different water inflows (lateral flows and rain water) could be used differentially by the two groups because evergreen oaks are known to have deeper roots than the deciduous (Lieth, 1975; Lopez‐Iglesias, Villar, & Poorter, 2014).

Additionally, in some Tyrrhenian plain forests, for example, there are documented cases with shallow perennial aquifers (Manes, Grignetti, Tinelli, Lenz, & Ciccioli, 1997; Mura & Rinaldi, 1986) able to sustain growth throughout the year. Because evergreen oaks experience a longer growing season (Baldocchi et al., 2010; Chabot & Hicks, 1982), the observed peak of co‐occurrence at high transpiration rates could be explained by temporal niche partitioning where mixtures of both groups increase the water consumption of the community by using water during different seasons similarly to other process already documents as for example increased light capture in a seasonal tropical tree experiment (Sapijanskas, Paquette, Potvin, Kunert, & Loreau, 2014). According to the classic coexistence theory (Chesson, 2000), temporal niches differences, for example arising from environmental fluctuation due to seasonality, are a fundamental stabilizing mechanism for coexistence: when a species is favored by the environment, the intra‐specific competition becomes stronger, while coexistence is enhanced with temporal niche differentiation. Overall, the model's assumptions of different water use and water stress tolerance between evergreen and deciduous oaks were based on yearly averages, and thus, the model has to be intended on yearly time scale, while a temporal niche partitioning should involve at least a seasonal time scale. Nevertheless, temporal niche partitioning could be relevant to improve the modeling capacity to explain oaks co‐occurrence.

Between the broad categories of deciduous and evergreens are the semi‐deciduous, defined as species having mean leaf life‐spans of 5–12 months (Villar & Merino, 2001). Literature reports that semi‐deciduous species are likely to share traits more closely with evergreen species that retain foliage for 2–3 years rather than with deciduous species having leaf life‐spans of 4–8 months (Reich, Walters, & Ellsworth, 1992; Villar & Merino, 2001). From our analysis, semi‐deciduous oaks showed evapotranspiration rates closer to those of evergreen species rather than to those of the deciduous (Figure 4c). The tests performed by including semi‐deciduous oaks either in the evergreen or in the deciduous group (Fig. S6) revealed that such species move the distribution of ET more toward low values than high values of ET, supporting the hypothesis that the semi‐deciduous are functionally more similar to the evergreens than to the deciduous. To our knowledge, this is the first large‐scale confirmation of the physiological proximity of the two groups.

Lastly, we also acknowledge several possible biases that should be taken into account. First, the observed species distribution has been influenced by anthropic perturbations (e.g. land use change, logging, and fire) and thus, forest composition might also not be at equilibrium. For example, Urbieta, Zavala, and Maranón (2008) reported that over the last few millennia, deciduous taxa of the Mediterranean region have been partially replaced by sclerophyllous species. However, anthropic perturbations may be difficult to detect at regional scale without detailed geo‐datasets describing land use history over the study area and over significant periods (i.e. decades to century).

Second, the model assumes that all the tree species in the same group share the same growth rate and transpiration function, whereas likely transpiration rates vary even among species within each group, suggesting that a more accurate test could be achieved with individual oaks species rather than functional groups. Indeed, different species within the same group may have distinct, albeit similar, physiologies, each yielding a slightly different value for the bounds in (5). This may lead PDFs of the ET to be generally broad, with large overlaps. However data from JRC are not enough for attempting an analysis at species level (i.e. considering single oaks). Similarly, field observations would represent the best way to investigate the hypothesis supported by the model. To our purpose we should have had field observations on oaks relative presence and, from the same plots, measure of soil water content or actual evapotranspiration, embracing several sites over a large extent of environmental conditions. The shortage of this kind of observations made the JRC and MODIS‐ET the most accurate and up‐to‐date large‐scale dataset suitable for our work.

Third, we cannot fail to take in consideration the uncertainties related to the spatial scales. For instance, working at such resolution (~1 km) some amount of small‐scale noise may be produced due to database inaccuracies and possible mismatches (see Section 3). Moreover, in our case, there is a probability that a grid cell could be classified as a “co‐occurrence” as deciduous and evergreen oaks are both present, though far apart. To minimize this probability, we selected grid cells where oaks presence exceeds 90%. Again, at 1 km of spatial resolution others minor environmental drivers are omitted. At this scale, the effect of slope, altitude and soil are implicitly included in the mean annual value of ET. Indeed, environmental heterogeneity could also explain why the PDFs of each group have wider distributions with respect to ET. Overall, uncertainty related to the scale is nevertheless important and never completely deniable, albeit addressed and minimized.

## 5 Conclusion

We believe that, because of its simplicity and clear‐cut assumptions, our coexistence model represents a useful tool for investigating the co‐occurrence of species having different water use and water stress tolerance, suggesting a simple (but not trivial) explanation. We used the model on a large extent assessment making care that the basic assumptions of the model were still valid at the spatial scale addressed; again, we moved under the spotlight a few cases that may signal some possible stabilizing mechanism for coexistence such as temporal niche partitioning, not accounted for in the model, that would allow to increase the understating of species interactions and, ultimately, improve the modeling capacity to explain co‐occurrence.

Higher resolution data, at least for some target areas, could be of help to better understand species interactions and further develop the model. Likewise, ecosystem gas exchange data, as obtained for instance from eddy covariance flux networks, could be helpful for the estimation of carbon and water balances between the different groups of vegetation. Overall, we hope that our contribution could also help scientific community to increase the application of the coexistence framework theory from small‐scale studies to landscape and regional scale.
