# Supplementary material for: Coexistence trend contingent to Mediterranean oaks with different leaf habits
Source: Ecol Evol. 2017 Mar 23;7(9):3006–15. doi: 10.1002/ece3.2840 (PMC5415544; doi:10.1002/ece3.2840)
Supplement: Supplementary file 1 [file ECE3-7-3006-s001.docx]

**SUPPORTING INFORMATION**

**Appendix S1.**

**Model derivation**

Here we show how the coexistence condition (5) of the main text can be deduced. For a full description of the model, and a complete study of its equilibria and their stability, please refer to (Di Paola et al., 2012)

A great deal of useful information can be extracted from the model embodied by equations (1-3) of the main text, just by assuming some reasonable monotonicity properties of the functions that appear in it. In particular, *F* is assumed to be a growing function of soil water content *W*; *H* needs to be a growing function of *W* only up to some intermediate value of water content, and may or may not be decreasing for larger water contents; *q , T_D_ , T_E_* are also growing functions of *W* .

Solving eq. (1) and (2) a *coexistence equilibrium* (i.e. both *D* and *E* ≠0) is reached when the following identity holds:

$$\frac{F\left( W_{C} \right)}{k_{D}}=\frac{H\left( W_{C} \right)}{k_{E}}=D_{C}+E_{C} \left( S1 \right)$$

Where *Wc, D_C_* and *E_C_* are the soil water content and the biomass density of the two species at the equilibrium of coexistence (signed by the subscript *C*), respectively. Solving eq.(4) of the main text with (S1), *D_C_* and *E_C_* can be expressed as

$D_{C}=\frac{k_{E}S\left( W_{C} \right)-T_{E}\left( W_{C} \right)H\left( W_{C} \right)}{k_{E}\left( T_{D}\left( W_{C} \right)-T_{E}\left( W_{C} \right) \right)},E_{c}=\frac{T_{D}\left( W_{c} \right)F\left( W_{c} \right)-k_{D}S\left( W_{c} \right)}{k_{D}\left( T_{D}\left( W_{C} \right)-T_{E}\left( W_{C} \right) \right)} \left( S2 \right)$

It can been proven (Di Paola et al., 2012) that the coexistence equilibrium is stable provided that the following conditions hold:

1. $T_{D}\left( W_{C} \right)>T_{E}\left( W_{C} \right)$;

2. $'\left( W_{C} \right)k_{E}>H'\left( W_{C} \right)k_{D}$ ;

3. A complicated inequality involving $H'\left( W_{C} \right),$which is always satisfied if

$H^{'}\left( W_{C} \right), {T^{'}}_{D}\left( W_{C} \right), {T^{'}}_{E}(W_{C})\geq0$;

where the prime denotes the derivative of the function. The first condition is that the deciduous species should transpire more than the evergreens; the second condition, together with (S1) if *k_D_* ≈ *k_E_*, translates into the statement that for lower-than-equilibrium water levels the growth rate of the evergreen should be higher than that of the deciduous, and the other way around at higher-than-equilibrium water levels; the third condition requires that the equilibrium happens at soil water contents which are not as high as to be detrimental to either group, in particular the evergreen. In addition to these physiologically reasonable requirements, the biomass density must be positive. From (S2), using (4) and (S1), the obvious constraint $D_{c},E_{c}>0$ turns into the inequalities:

$T_{E}\left( W_{C} \right)\left( W_{C} \right)\left( D_{C}+E_{C} \right)+e<p-q\left( W_{C} \right)<T_{D}\left( W_{C} \right){(D}_{C}+E_{C})+e$ (S3)

At the left and right extremes of this chain of inequalities are the evapotranspiration rates of theoretical forests (i.e. that would never be observed) having the same total biomass density as the forest in which coexistence occurs, but contain (and thus have the transpiration rate of) only one of the two groups of species. In the centre we find the hydrological balance between precipitation and water losses. In equilibrium conditions the hydrological balance equals the evapotranspiration of the forest in which coexistence occurs. It can also be proven (Di Paola et al., 2012) that, in the model, as long as (5) is satisfied, the equilibrium soil water content *W_C_* remains constant

We observe that in (S3) neither the equilibrium soil water content, nor the total equilibrium biomass depend on the abiotic hydrological parameters, thus we conclude that variations in the precipitation *p* may only reflect into the relative abundance of the two groups of coexisting species. In particular, using (S1) in (S3), we have that if *p* is as low as to make $p-q\left( W_{C} \right)=T_{E}\left( W_{C} \right)\left( W_{C} \right)H\left( W_{C} \right)/{k_{E}}\left( W_{C} \right)/{k_{E}}$ then all the biomass is composed by evergreen; conversely, if *p* is as high as to make $p-q\left( W_{C} \right)=T_{D}\left( W_{C} \right)F\left( W_{C} \right)/{k_{D}}$, then all the biomass is composed of deciduous.

The soil water content of the equilibria containing only either deciduous or evergreen species (respectively, *W_D_* and *W_E_*) are given by the values that satisfy the following equations:

$$p-q\left( W_{D} \right)=T_{D}\left( W_{D} \right)\frac{F\left( W_{D} \right)}{k_{D}}+e, stable forW_{D}>W_{C} \left( S4 \right)$$

$$p-q\left( W_{E} \right)=T_{E}\left( W_{E} \right)\frac{H\left( W_{E} \right)}{k_{E}}+e, stable for W_{E}<W_{C} \left( S5 \right)$$

From (1) and (2) it follows that the quantities $F(W_{D})/k_{D}$ and $H(W_{E})/k_{E}$ are the biomass densities of these equilibria, of which the first is larger and the second is smaller than the biomass density of the coexistence equilibrium. Therefore, the right-hand side of equations (S4) and (S5) represent the actual evapotranspiration of forests containing only one group of species.

When the hydrological balance of precipitation minus percolation is not between the two extreme levels of evapotranspiration, as prescribed by (S3), the model predicts that one of the two groups of species eventually disappears. If the soil water content becomes higher than *W_C_* then only the deciduous survive. Conversely, a decrease in soil water content below *W_C_* causes the deciduous to become extinct. Because *F*, *T_D_* , *T_E_*, *q*, and (up at least to *W_C_*) *H* are growing functions of *W*, then from (S1), (S4) and (S5) it follows that a stable forest of evergreen must have an evapotranspiration smaller than that of a stable forest with coexistence of both groups of species, which, in turn, must be smaller than the evapotranspiration of a forest of deciduous, thus proving the validity of the inequality (5) reported in the main text of the paper.

**Appendix S2**

Let (*d_1_*,*d_2_*,...,*d_N_*) be a set of data (e.g. the MODIS ET data) distributed according to an unknown probability. The probability density function estimated from data evaluated at the specified value *d* is:

$$f\left( d \right)=\frac{\sum_{i=1}^{N} g_{h}\left( {d-d}_{i} \right)}{N} \left( S6 \right)$$

Where $g_{h}$is the is the Gaussian distribution having variance equal to *h^2^*.

$$g_{h}\left( x \right)=\frac{1}{h\sqrt{2\pi}}exp\left( -\frac{1}{2}\frac{x^{2}}{h^{2}} \right)$$

If Gaussian basis functions are used to approximate univariate data, and the underlying density being estimated is Gaussian then it can be shown that the optimal choice for *h* is

$$h=\left( \frac{4\sigma^{5}}{3N} \right)^{\frac{1}{5}}1.06\sigma N^{{-1}/5}$$

where *N* is the number of data points and ? is the standard deviation of the samples. If the data are not Gaussian, usually the above formula still yields a reasonable estimate of the PDF of the data (Silverman, 1986; Bowman & Azzalini, 1997).

**Supporting Figures**


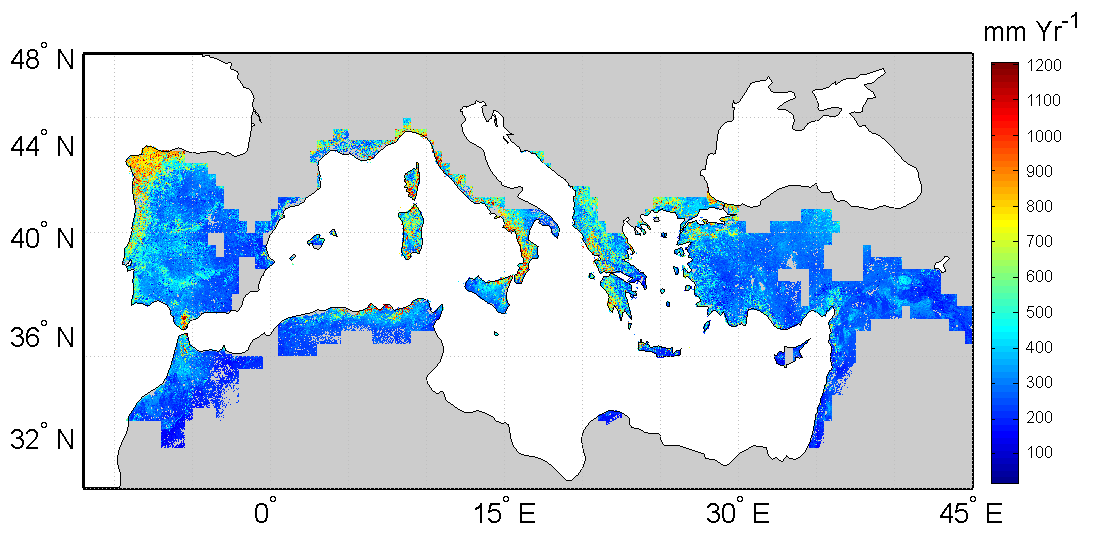


**Figure S1**. Mean annual evapotranspiration (ET) derived from MODIS products for the period 2000-2011. Data are highlighted only for the Mediterranean climate type (as defined by (Köttek et al., 2006). The ET ranges approximately between 0 and 1800mmYr^-1^, here we report the range 0-1200mmY^-1^ to better display colors. This and all the following maps use the Gall-Peters projection.


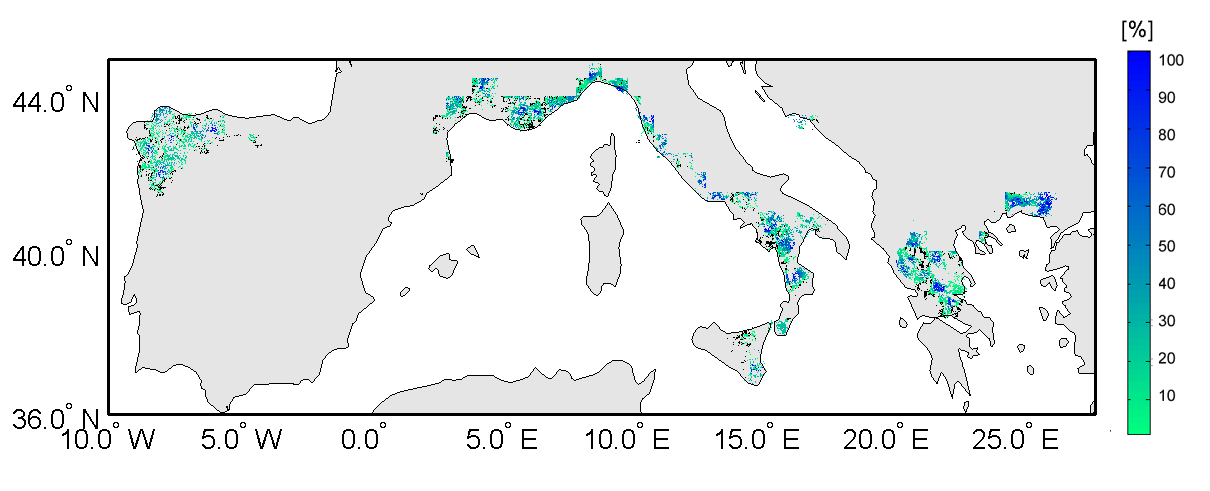


**Figure S2**. Distribution of deciduous oaks (*Q. cerris*, *Q. frainetto*, *Q. Pubescens*, *Q. petraea* and *Q. robur*) expressed as percentage of land cover.


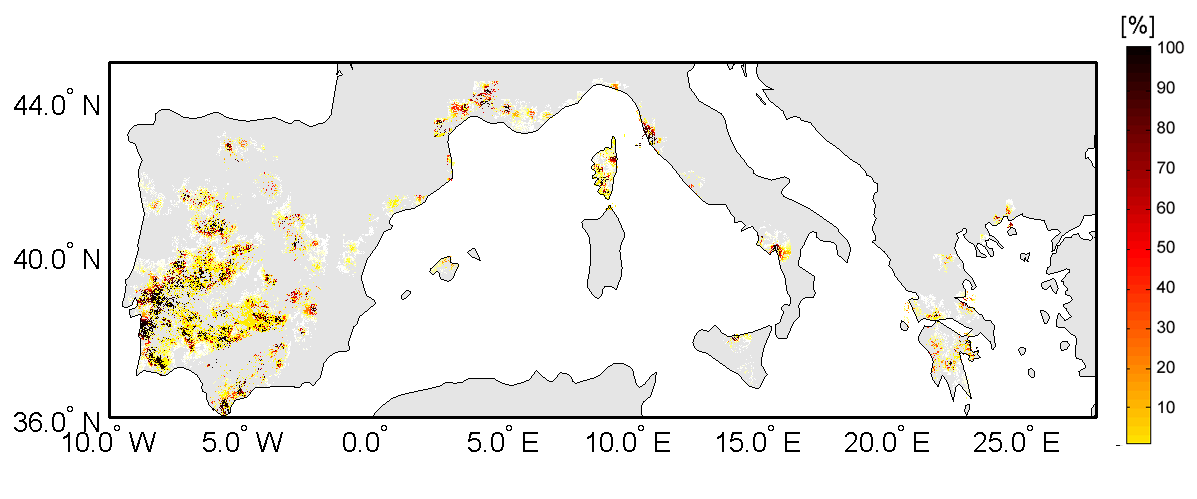


**Figure S3**. Distribution of evergreen oaks (*Q. ilex*, *Q. coccifera*, *Q. suber*, and *Q. rotundifolia*) expressed as percentage of land cover. Map designed in Gall-Peters projection.


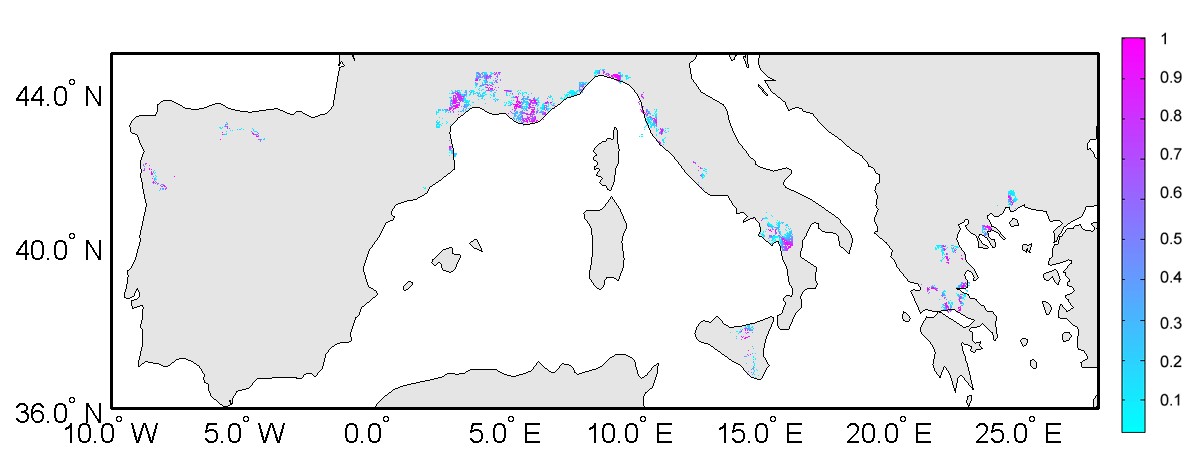


**Figure S4**. Sites of co-occurrence of evergreen and deciduous oaks in the Mediterranean climate zone. The max value 1 represents the case where there is equal occurrence (50%-50%) of the two groups, while for CO=0 there is a total dominance of one of the two groups.


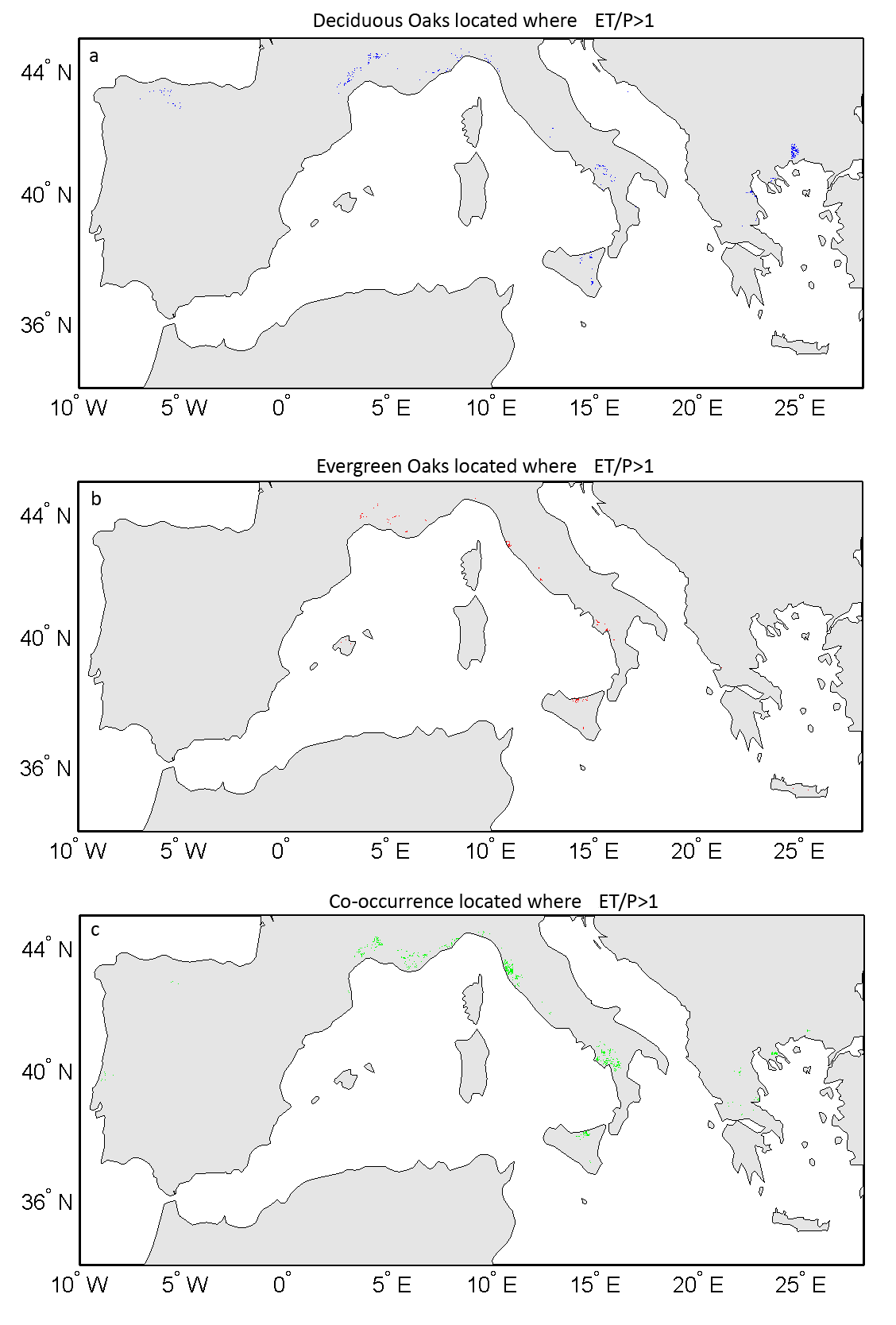


**Figure S5.** Geo-locations of data (cover>90%) having ratio ET/P>1. a) deciduous group; b) evergreen group c) Cases of co-occurrence.


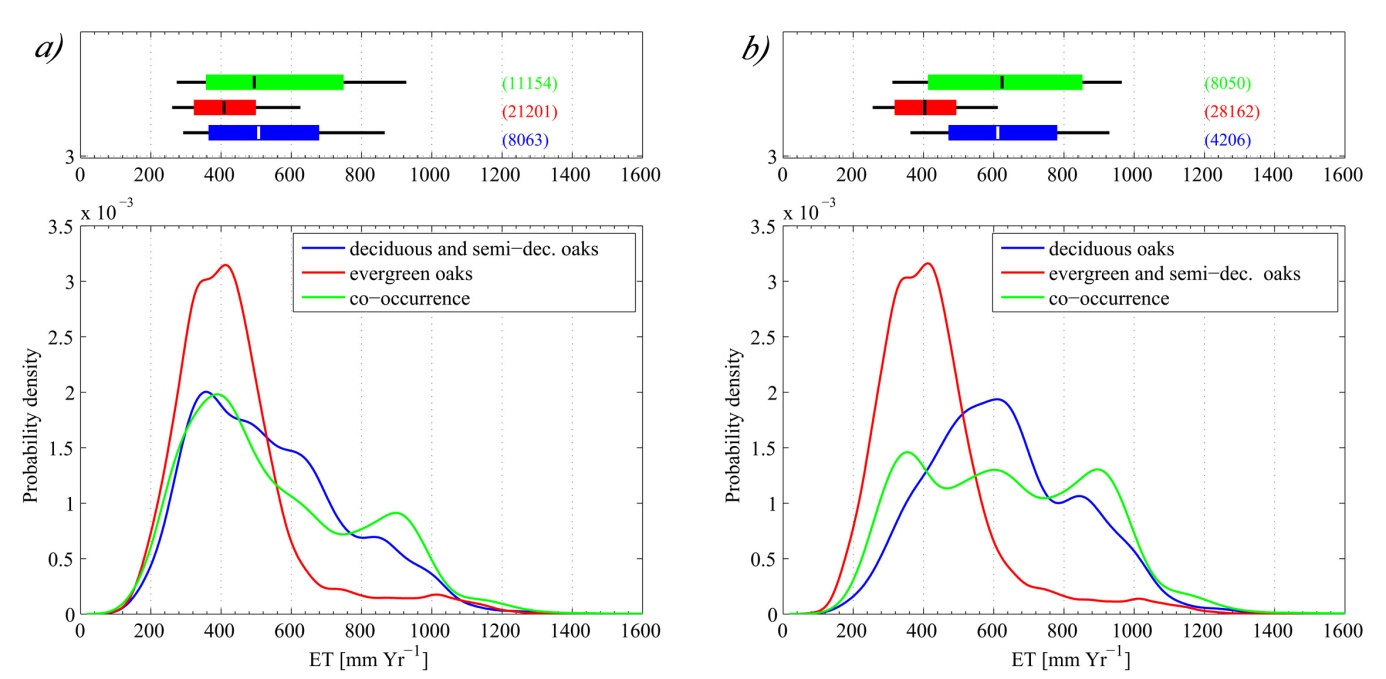


**Figure S6**. As Figure 3*a* but including data of semi-deciduous into the group of *a)* deciduous oaks; *b)* evergreen oaks. Probability density functions estimated from data through eq. (12);


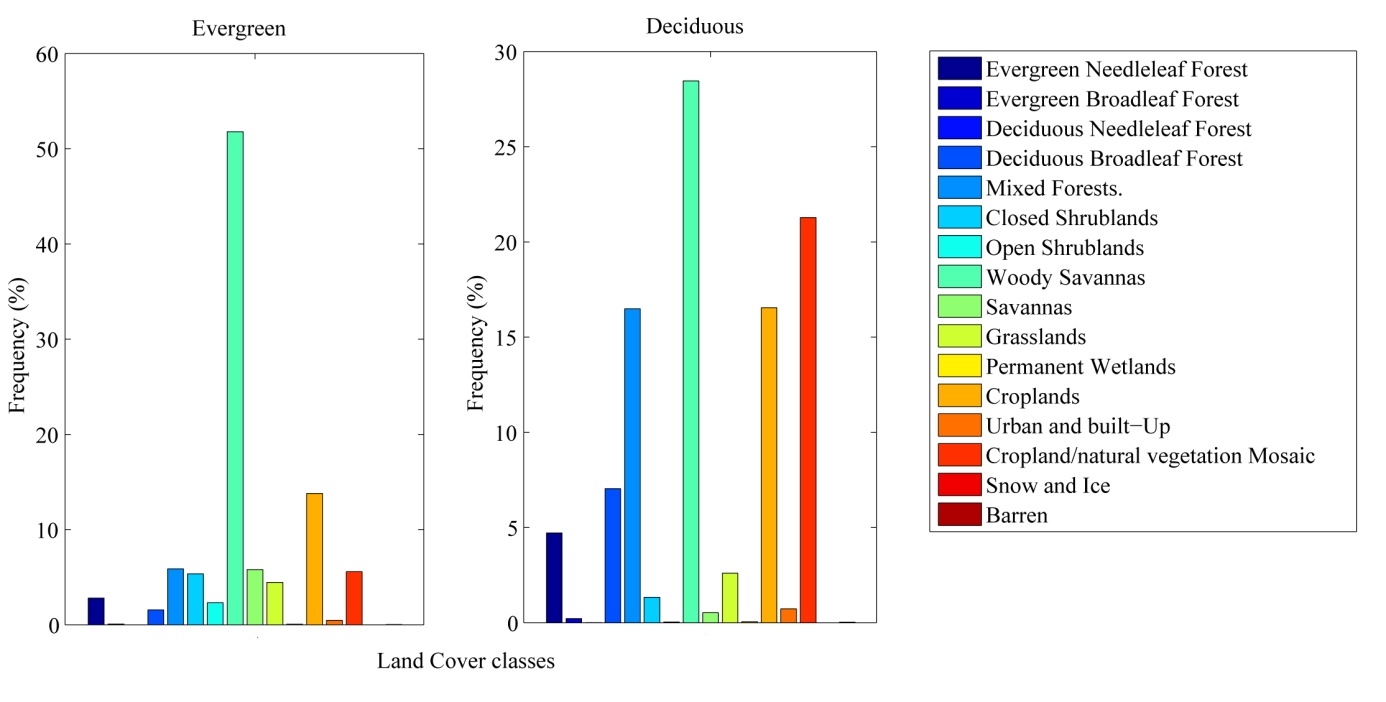


**Figure S7.** Land cover classes (according to the MODIS Land Cover data set (Friedl et al., 2002) of data distributions of evergreen and deciduous oaks. The occurrences are expressed as percentage on the total pixels.

**Supporting Table**

**Table S1**. Descriptive statistic of Mean Annual Evapotranspiration (ET) for the evergreen group, deciduous group and cases of co-occurrence of both of them: median, interquartile range (IQR, equal to 75^th^-25^th^ percentiles), probability of presence (*P*) below and over the thresholds of 280mmYr^-1^ and 870mmYr^-1^, respectively.

**Median (mmYr^-1^)** **IQR (mmYr^-1^)**  ***P*(ET<280) *P*(ET>870)**

*Fig. 2a- ET*

Evergreen 404 166 13.5 4.1

Co-occurrence 684 385 3.9 27.7

Deciduous 614 302 2.5 15.2

*Fig. 2b - as Fig. 3a but excluding data points with ET/P>1*

Evergreen 391 148 14.7 <0.01

Co-occurrence 536 241 6.6 <0.01

Deciduous 540 209 3.5 4.0

*Fig. S6a – as Fig.3 but including data of semi-deciduous into the group of deciduous oaks*

Evergreen 408 168 13.0 4.4

Co-occurrence 492 384 10.3 15.5

Deciduous 505 306 7.5 9.2

*Fig. S6b – as Fig.3 but including data of semi-deciduous into the group of evergreen oaks*

Evergreen 403 166 14.2 3.4

Co-occurrence 622 435 5.2 22.4

Deciduous 611 301 2.6 14.8

**Table S2.** Results of the One-tailed Mann-Whitney U-test and Kolmogorov-Smirnov (KS) tests comparing Potential Evapotranspiration (PET) and Mean Annual Evapotranspiration (ET) of Evergreen (E), Deciduous (D) and co-occurrence (CO) of both the groups under different assumptions.. For each test we report the number of data points (N), the statistics and the p-value. For the Mann-Whitney test, *p* is the probability of the null-hypothesis which assumes that the central tendency of the first group (namely, E, E, CO for, respectively, the first, second and third column) is higher or equal than that of the second group (D, CO, D for the first, second, third column). If an asterisk (*) appears next to the p-value, then the null-hypothesis assumes that the first group has a central tendency lower or equal than that of the first.

***E vs D E vs. CO CO vs. D***

*Fig 1 – PET*

N (205916 and 74193)

*U* **3.4 10^10^*

*p* *<10^-15^*

*KS 0.55*

*p <10^-15^*

*Fig. 2a - ET*

N (22690 and 4361) (22690 and 6157) (6157 and 4361)

*U 2.7 10^8^ 2.8 10^8^ 3.4 10^7^*

*p <10^-15^ <10^-15^ <10^-15^(*)*

*KS 0.49 0.53 0.15*

*p* *<10^-15^* <*10^-15^* *<10^-15^*

*Fig. 2b - as Fig. 3a but excluding data points with ET/P>1*

N (20609 and 3131) (20609 and 3654) (20609 and 3131)

*U 2.2 10^8^ 2.3 10^8^ 1.2 10^7^*

*p <10^-15^ <10^-15^ 1.0 10^-5^*

*KS 0.44 0.42 0.07*

*p* *<10^-15^* <*10^-15^* *1.1 10^-7^*

*Fig. S6a – As Fig.3a but including data of semi-deciduous into the group of deciduous oaks*

N (21201 and 8063) (21201 and 11154) (11154 and 8063)

*U 2.8 10 ^8^ 3.1 10^8^  1.0 10^8^*

*p <10^-15^  <10^-15^  0.09(*)*

*KS 0.28 0.28 0.07*

*p* *<10^-15^* <*10^-15^* *<10^-15^*

*Fig. S6b – As Fig.3a but including data of semi-deciduous into the group of evergreen oaks*

N (28162 and 4206) (28162 and 8050) (8050 and 4206)

*U 4.2 10^8^ 4.5 10^10^ 4.9 10^7^*

*p <10^-15^  <10^-15^ 0.20(*)*

*KS 0.48 0.45 0.09*

*p* *<10^-15^*  <*10^-15^* *<10^-15^*

**SI References**

Bowman, A. W., & Azzalini, A. (1997). Applied smoothing techniques for data analysis: the kernel approach with S-Plus illustrations: the kernel approach with S-Plus illustrations. Oxford University Press.

Di Paola, A., Valentini, R., & Paparella, F. (2012). Climate Change Threatens Coexistence within Communities of Mediterranean Forested Wetlands. PloS one, 7(10), e44727

Friedl, M. A., McIver, D. K., Hodges, J. C., Zhang, X. Y., Muchoney, D., Strahler, A. H., ... & Schaaf, C. (2002). Global land cover mapping from MODIS: algorithms and early results. *Remote Sensing of Environment*, 83(1), 287-302.

Kottek, M., Grieser, J., Beck, C., Rudolf, B., & Rubel, F. (2006). World map of the Köppen-Geiger climate classification updated. Meteorologische Zeitschrift, 15(3), 259-263

Silverman, B. W. (1986). Density estimation for statistics and data analysis (Vol. 26). CRC press.
